# Influence of plasma exosomes from women living with HIV Stratified by HAND on monocyte subpopulations from healthy women without HIV

**DOI:** 10.1007/s13365-024-01240-9

**Published:** 2025-01-30

**Authors:** Bryan Jael Collazo, Lorivette Ortiz-Valentín, Cristhian G. Negrón-Rodríguez, Juan Carlos Medina-Colón, Yisel M. Cantres-Rosario, Elaine Rodríguez, Valerie Wojna, Yamil Gerena

**Affiliations:** 1 Department of Pharmacology and Toxicology, Medical Sciences Campus, University of Puerto Rico, San Juan, 00936-5067 Puerto Rico; 2 Department of Microbiology and Medical Zoology, Medical Sciences Campus, University of Puerto Rico, San Juan, 00936-5067 Puerto Rico; 3 Neurology Division, Medical Sciences Campus, University of Puerto Rico, San Juan, 00936-5067 Puerto Rico

**Keywords:** HIV-positive exosomes, CD14/CD16 monocytes, HAND

## Abstract

The role of plasma exosomes from people living with HIV (PLWH) with HAND in the phenotypic profile of uninfected monocytes remains unknown. We hypothesized that these exosomes influence the CD14/CD16 phenotypical profile of uninfected monocytes in a time-dependent manner. Exosomes were collected via ultracentrifugation from the plasma of women living with HIV (WLWH) and healthy controls stratified according to their cognition into normal cognition (NC) or symptomatic neurocognitive impairment (SNI) groups. Monocyte subsets were identified via flow cytometry by using anti-CD14 and anti-CD16 fluorescent antibodies. Exosome uptake and changes in the percentages of monocyte subpopulations were analyzed from 1 to 24 h. The following results were obtained. (1) The uptake of HIV-negative exosomes by total uninfected monocytes was observed at 24 h, whereas the uptake of HIV-positive exosomes was observed at an earlier time point at 6 h. (2) HIV-positive exosomes significantly decreased the percentage of classical monocytes and increased intermediate and nonclassical monocytes at 24 h. (3) The uptake of NC exosomes was observed at an early time point at 6 h compared with SNI in all of the monocyte subsets. (4) Higher percentages of monocyte subsets were observed when cells were exposed to NC exosomes at 1 h, 6 h, or 24 h than when monocytes were exposed to exosomes from SNI patients. Our findings may help to identify new targets and molecular mechanisms that are involved in the pathogenesis of HAND.

## Introduction

HIV-associated neurocognitive disorders (HAND) continue to be a major cause of morbidity and mortality in PLWH (Heaton et al. 2010; Letendre et al. 2010; Vivithanaporn et al. 2010). Despite the success of antiretroviral therapy (ART) in improving the life expectancies of PLWH, the prevalence of HAND in milder forms is currently estimated at 20–50% (Eggers et al. 2017; Heaton et al. 2010; Letendre et al. 2010; Sacktor et al. 2016; Saylor et al. 2016; Simioni et al. 2010; Vivithanaporn et al. 2010). The pathophysiology of HAND involves increased blood–brain barrier (BBB) permeability, persistence of monocyte activation, systemic and central nervous system (CNS) inflammation, oxidative stress, neuronal dysfunction, and cell death (Cantres-Rosario et al. 2013; Gannon et al. 2011; Liner et al. 2010; McArthur et al. 2010). Currently, the associations of altered proportions of monocyte subpopulations based on the expression of CD14/CD16 surface markers with HIV neuropathogenesis remain to be investigated.

Studies have shown that despite ART, the number of inflammatory activated CD14 + CD16 + monocyte subsets increases in the peripheral blood, and these subsets cross the BBB and infect CNS macrophages and microglia, thus contributing to the development of HAND and the brain's viral reservoir (Buckner et al. 2011; Campbell et al. 2014; Fischer-Smith 2001; Ndhlovu et al. 2015; Valcour et al. 2011; Veenstra et al. 2017, 2019; Williams et al. 2014; Williams et al. 2012, 2013; Wong et al. 2019). However, the phenotype of the monocyte subpopulation that migrates into the brain and that is associated with HAND remains to be clarified.

Human monocytes are subdivided into three subsets based on the surface expression of CD14 and CD16: classical (CD14 + + CD16-), proinflammatory intermediate (CD14 + + CD16 +) and “patrolling” nonclassical (CD14 + CD16 +) subsets, with each subset representing approximately 90%, 5%, and 5% of the total circulating monocytes, respectively (Ożańska et al. 2020). Studies using grafted human classical monocytes in a humanized mouse model revealed that this subpopulation sequentially differentiates into intermediate and nonclassical monocyte subpopulations (Patel et al. 2017). Variations in the percentages of monocyte subpopulations can be observed in inflammatory diseases and infections. For example, a previous study in patients with sepsis or chronic inflammatory disease revealed that classical monocytes exhibit phagocytic functions without inflammatory attributes, intermediate monocytes display both phagocytic and inflammatory responses, and nonclassical monocytes exhibit inflammatory characteristics upon activation (Mukherjee et al. 2015). Furthermore, the levels of intermediate and nonclassical monocytes are increased in patients with sepsis, and only the nonclassical subpopulation is elevated in patients with chronic inflammatory diseases ((Mukherjee et al. 2015).

Alterations in monocyte subsets have also been observed in PLWH with HAND. The CD14 + CD16 + monocyte subpopulation is critical in the neuropathogenesis of HIV infection, and the proportion of this subpopulation can be increased to ∼40% in PLWH associated with HAND, despite ART (Campbell et al. 2014; Valcour et al. 2011; Williams et al. 2014; Williams et al. 2013). The factors that are responsible for the persistent changes in the monocyte subset phenotypic profiles of virologically suppressed PLWH are not clearly understood. Exosomes have emerged as being possible mediators of immune system activation (Chettimada et al. 2018; Li et al. 2019). These structures are membranous nanovesicles (with a size range of ~ 40–160 nm) of endosomal origin that are released from most cells and that are recognized as being mediators of cellular communication in normal physiological processes, such as the balance of the immune response, regulation of central and peripheral immunity, and cellular differentiation (Kalluri and LeBleu 2020; McKelvey et al. 2015; Saeedi et al. 2019; Ståhl et al. 2019). Exosomes can play a key role in different pathological conditions, including those linked to long-term inflammation associated with chronic immune activation, such as HIV infection and neurological diseases (Pérez et al. 2019; Ståhl et al. 2019). Exosomes may bind to receptors on target cells, thereby transducing a signal or transferring functional receptors, proteins, lipids, DNA, mRNAs, or miRNAs from parent cells to recipient cells, thus affecting cell function and behavior (Ståhl et al. 2019). For example, Freeman et al. revealed that monocyte subpopulations from female donors without allergies were able to internalize exosomes collected from the plasma of individuals with type 2 diabetes mellitus, thus inducing alterations in monocytic function, including increased expression of genes associated with immune function and inflammation, apoptosis and oxidative stress (Freeman et al. 2018). Moreover, studies have reported that exosomes are more abundant in the plasma of PLWH than in healthy controls (Pérez et al. 2019). Chettimada et al. showed that plasma exosomes from ART-treated PLWH containing immune activation markers such as CD14, C-reactive protein (CRP), human leukocyte antigen-A (HLA-A), and HLA-B were internalized by the human acute monocytic leukemia cell line THP-1 and induced the expression of genes associated with interferon responses, innate immune responses, and inflammation (Chettimada et al. 2018).

Previously, we reported that exosomes isolated from the plasma of WLWH with HAND in our Hispanic/Latino Longitudinal Cohort (HLLC) exhibited significantly increased levels of soluble insulin receptor (sIR), HIV-1 Tat, and ROS compared with those in exosomes isolated from the plasma of healthy controls (Cantres-Rosario et al. 2022). However, the influence of these exosomes on the phenotypic changes of HIV-uninfected monocytes has never been explored. In this study, we investigated whether the phenotypic profile of monocytes from healthy women without HIV was influenced by the uptake of plasma exosomes collected from WLWH stratified by HAND.

## Methods

### Participants and study design

This was a retrospective cross-sectional study nested in the Hispanic/Latino Longitudinal Cohort of Women (HLLC) that used patient database information and an exosome sample repository. This study was approved by the University of Puerto Rico Medical Sciences Campus (UPR, MSC) Institutional Review Board, and all of the participants provided written informed consent. The HLLC is a unique cohort of Hispanic WLWH who were longitudinally characterized with viral and immune profiles, neurological exams, and neuropsychological tests. Ten (10) WLWH without a history of diabetes and five (5) healthy controls were evaluated as previously described (Gerena et al. 2012, 2015; Wojna et al. 2006, 2007). WLWH were grouped according to their cognitive performance as having normal cognition (NC; *n* = 5) or symptomatic impairment (SNI; *n* = 5). The characteristics of these patients are detailed in Table 1. Education level was self-reported and verified via the vocabulary subtest of the Wechsler Adult Intelligent Test and the reading subtest modality of the Woodcock-Muñoz Test. No significant differences were observed between the groups in terms of age, education, CD4 cell count, plasma viral load, hepatitis C virus coinfection, body mass index, toxicology, or antiretroviral therapy (Table 1). In addition, three (3) healthy women without HIV (28 ± 3-years-old) were recruited to serve as PBMC donors to be exposed to the exosomes of the HLLC.

**Table 1:** Characteristics of HIV-negative and HIV + women

	HIV-negative controls	HIV +	*p*-value	Normal Cognition (NC)	Symptomatic Neurocognitive Impairment (SNI)	*p*-value
	*n* = 5	*n* = 10		*n* = 5	*n* = 5	
Age (yrs.)	45.00 (28.50, 54.00)	42.00 (32.75, 50.50)	> 0.999	36.00 (32.50, 53.50)	42.00 (36.50, 50.00)	> 0.999
Education (level)	14.00 (12.00, 17.50)	12.50 (12.00, 14.50)	0.479	14.00 (12.00, 16.00)	12.00 (10.50, 13.50)	0.286
Total CD4 (cells/mm^3^)		644.00 (475.00, 804.00)		616.00 (267.50, 971.50)	669.00 (484.00, 884.00)	0.691
Plasma HIV RNA (Log copies/mL)		4.45 (1.30, 4.66)		1.30 (1.30, 5.34)	4.46 (2.09, 4.61)	0.914
Hepatitis C Virus Positive n (%)	0 (0%)	3 (30%)	0.279	1 (20%)	2 (40%)	0.490
Body Mass Index	34.25 (26.63, 43.05)	26.05 (22.93, 33.48)	0.374	24.40 (21.35, 30.00)	29.30 (24.10, 36.15)	0.310
Toxicology Positive n (%) Marijuana (n) Cocaine (n) Both (n) Unspecified (n)	0 (0%) 0 0 0 0	2 (20%) 0 1 1 0	0.283	1 (20%) 0 0 1 0	1 (20%) 0 1 0 0	1.000
ART n (%)		10 (100%)		5 (100%)	5 (100%)	

### Isolation of PBMCs from healthy women without HIV

Fresh blood samples from three (3) different healthy female donors without HIV were collected in acid citrate dextrose (ACD) tubes. PBMCs were isolated via Ficoll-Hypaque density gradient centrifugation (Pfizer, Inc., New York, NY). Subsequently, the PBMCs were cultured (5 × 10^5^ cells) in RPMI-1640 (HyClone, Logan, UT) supplemented with 10% fetal bovine serum (FBS) at 37 °C and 5% CO_2_. PBMCs were exposed to exosomes from WLWH (NC and SNI) and from healthy women of the HLLC (as controls) for investigations related to monocytic exosome uptake and phenotypic subpopulation profiles, as described below.

### Exosomal membrane labeling and exosome uptake assay

The membranes of plasma exosomes from WLWH (NC and SNI) and healthy controls of the HLLC were labeled by using the PKH-67 Green Fluorescent Cell Linker Kit, based on the manufacturer’s instructions (Sigma Aldrich). PKH-67-labeled exosomes (20 μg) were added to cultured PBMCs from healthy female donors without HIV, and exosome uptake by monocytes was analyzed at different times (1, 6, and 24 h). After incubation, the cells were collected and centrifuged at 1,200 rpm for 5 min at 20 °C. PBMCs were then stained with anti-human CD3 (PerCPCy5.5), anti-human CD14 (PE/Cy7), and CD16 (Pacific Blue) antibodies. Exosome uptake levels and changes in the percentages of CD14/CD16 monocyte subpopulations were analyzed via flow cytometry. Cell viability was monitored via the use of a zombie green amine-reactive fluorescent dye dilution (1:1000) for 15 min at room temperature to label the DNA of the dead cells. H_2_O_2_ (500 μM) was used as a positive control for apoptosis.

### Flow cytometry

All of the flow cytometric analyses were performed by using a FACSCelesta flow cytometer (BD Biosciences, San Jose, CA). Monocyte subsets were identified based on CD14 and CD16 expression (classical, nonclassical, and intermediate subsets) and after incubation with patient exosomes. The exclusion of CD3 lymphocytes was performed via the use of a specific anti-CD3 fluorescent antibody. FACSDiva software (BD Biosciences, San Jose, CA) was used for data acquisition and multivariate analysis. PKH-67 Green Fluorescent (bandpass filter: 530/30 nm), PerCPCy5.5 (bandpass filter: 695/40 nm), PE/Cy7 (bandpass filter: 780/60 nm), and Pacific Blue (bandpass filter: 450/40 nm) emissions were measured via excitation with blue (488 nm) or violet (405 nm) lasers, respectively. Data on the scatter parameters and histograms were acquired in log mode. One hundred thousand events were evaluated for each sample, and the median peak channel obtained from the histograms was used to determine the level of exosome uptake. FlowJo software was used for all of the cytometric analyses (FlowJo LLC, Ashland, OR).

### Statistical analysis

We compared exosome uptake levels and changes in monocyte subpopulations across different plasma exosome groups via triplicate assays averaged for each PBMC donor. The results are expressed as the mean ± standard error of the mean (SEM). Due to the fact that the data were not normally distributed, these comparisons were made via nonparametric associations (Kruskal–Wallis or Mann–Whitney tests). All of the statistical analyses were performed via Prism version 8.0.1 (GraphPad, La Jolla, CA) for Windows. A *p* value < 0.05 was considered to be statistically significant.

## Results

### Differences in the uptake of HIV-positive and HIV-negative plasma exosomes by monocytes

**Fig. 1:**
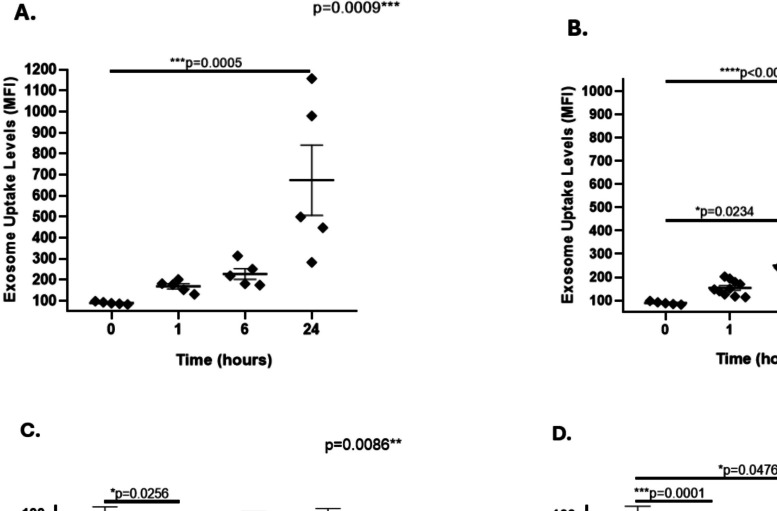
Uptake of HIV-negative and HIV-positive plasma exosomes by total monocytes. PBMCs from healthy female were incubated with exosomes from healthy female controls or WLWH for different durations and exosome uptake and cell viability in total monocytes were monitored via flow cytometry. **A** The exosome uptake significantly increased in monocytes exposed to control exosomes at 24 h (***p = 0.0005) than in those at 0 h. **B** The exosome uptake levels significantly increased in monocytes exposed to HIV-positive exosomes at 6 h (*p = 0.0234) or 24 h (****p < 0.0001) than in those at 0 h. **C** No significant differences were observed in the percentage of live monocytes exposed to HIV-negative or HIV-positive exosomes at 6 h than in those untreated (UT), whereas a significant decrease (*p = 0.0256) was observed when cells were exposed to H_2_O_2_. **D** The percentage of live monocytes significantly decreased in monocytes exposed to HIV-positive exosomes at 24 h (*p = 0.0476) or H2O2 (***p = 0.0001) than in those untreated. Data were expressed as mean ± SEM. Each point on the graph represents the average of 3 assays. Vertical lines at each bar represent SEM. The bars on the graph represent the average of 3 assays. *p < 0.05, ***p < 0.001, ****p < 0.0001; MFI = Median Fluorescence Intensity. The *p*-value illustrated in the right corner of each panel represents the ANOVA test value for the comparison of the four groups

PBMCs from healthy female donors were incubated with exosomes from WLWH or healthy female controls from the HLLC for different durations (1 h, 6 h, or 24 h), and exosome uptake and cell viability in total monocytes were monitored via flow cytometry. Our initial results revealed a significant increase in exosome uptake by total monocytes exposed to exosomes from healthy female controls at 24 h (Fig. 1A). However, the monocytic uptake of exosomes collected from WLWH significantly increased at both 6 and 24 h (Fig. 1B). When we compared the exosome uptake levels between HIV-negative and HIV-positive exosomes at 1, 6, or 24 h, no significant differences were observed. Compared with that of untreated cells, the viability of total monocytes exposed to either HIV-negative or HIV-positive exosomes was not affected at 6 h (Fig. 1C); however, a significant decrease in viability was observed in both groups at 24 h (Fig. 1D).

**Fig. 2:**
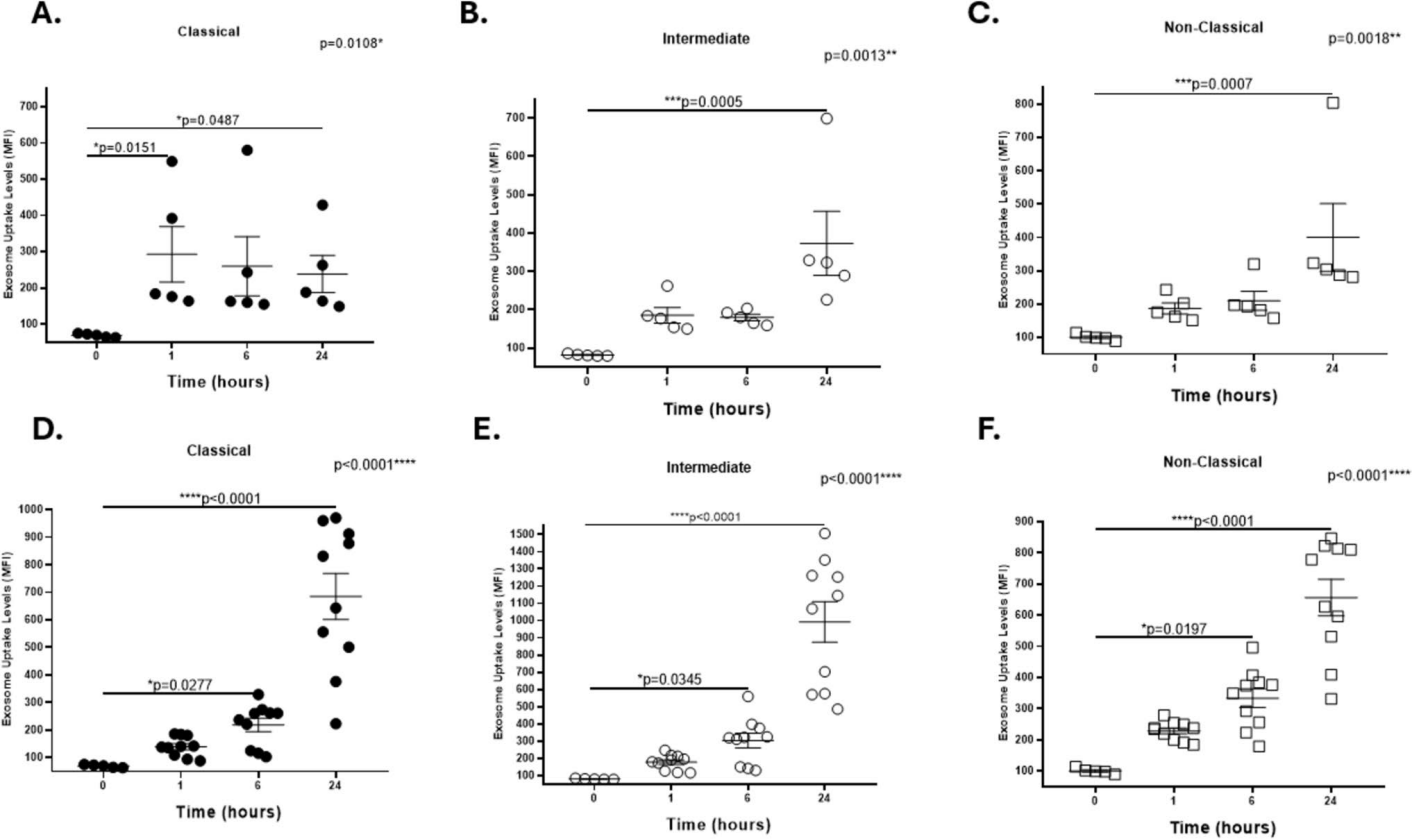
Uptake of HIV-negative and HIV-positive plasma exosomes by CD16/CD16 monocyte subsets. PBMCs from healthy female donors were incubated with exosomes from healthy female controls (panels **A**, **B**, **C**) or WLWH (panels **D**, **E**, **F**) and exosome uptake in monocyte subpopulations were monitored via flow cytometry. **A** The exosome uptake levels significantly increased in classical monocytes exposed to control exosomes at 1 h (*p = 0.0151) and 24 h (*p = 0.0487) than in those at 0 h. However, intermediate, and non-classical monocytes significantly internalized control exosomes at 24 h (**p = 0.0005 and *** *p* = 0.0007, respectively) (**B**, **C**). Exosome uptake significantly increased in classical, intermediate, and non-classical monocytes exposed to HIV-positive exosomes at 6 h (*p = 0.0277, * *p* = 0.0345, and * *p* = 0.0197, respectively) or 24 h (****p < 0.0001, ****p < 0.0001, and ****p < 0.0001, respectively) than in those at 0 h (**D**, **E**, **F**). Data were expressed as mean ± SEM. Each point on the graph represents the average of 3 assays. *p < 0.05, **p < 0.01, ***p < 0.001, ****p < 0.0001; MFI = Median Fluorescence Intensity. The *p*-value illustrated in the right corner of each panel represents the ANOVA test value for the comparison of the four groups

When we analyzed exosome uptake in different monocyte subpopulations, a significant increase was observed in classical monocytes exposed to HIV-negative exosomes at 1 and 24 h (Fig. 2A), whereas intermediate and nonclassical monocytes were significantly internalized only at 24 h (Fig. 2B, C). In contrast, a significant increase in exosome uptake was observed in all of the monocyte subsets at 6 and 24 h when they were exposed to exosomes from WLWH (Fig. 2D, E, F).

When comparing exosome uptake levels between monocytes exposed to HIV-negative and HIV-positive exosomes at 1, 6, or 24 h, we observed a significant decrease in classical monocytes at 1 h (*p* = 0.0300) and a significant increase at 24 h (*p* = 0.0047). For the intermediate subpopulation, a significant increase was observed at 24 h (p = 0.0047). In addition, exosome uptake was significantly greater in nonclassical monocytes exposed to HIV-positive exosomes at 6 h (*p* = 0.0280) and at 24 h (*p* = 0.0193) (Table 2). Exosome uptake levels did not change in monocyte subpopulations exposed to HIV-negative exosomes; however, they were significantly greater in nonclassical monocytes than in classical monocytes when exposed to HIV-positive exosomes at 1 h (*p* = 0.0003) and 6 h (*p* = 0.0443).

**Table 2:** Exosome uptake levels in monocyte subpopulations exposed to HIV-positive and HIV-negative exosomes at different times

Monocytes Subpopulations	Time	HIV – Exosome Uptake **(MFI ± SEM)**	HIV + Exosome Uptake **(MFI ± SEM)**	*P*-*V*alue
Classical	1 h 6 h 24 h	293.0 ± 76.66 260.2 ± 81.59 238.6 ± 51.48	139.9 ± 11.31 218.8 ± 24.41 684.9 ± 83.52	*p = 0.0300 *p* = 0.9530 *p = 0.0047
Intermediate	1 h 6 h 24 h	185.4 ± 20.22 179.8 ± 8.18 373.2 ± 83.48	178.6 ± 14.15 304.3 ± 42.16 992.6 ± 117.9	*p* = > 0.9999 *p* = 0.2544 *p = 0.0047
Non-Classical	1 h 6 h 24 h	186.6 ± 16.47 210.0 ± 28.32 399.8 ± 101.3	228.4 ± 9.47 333.6 ± 30.16 656.3 ± 59.03	*p* = 0.0703 *p = 0.0280 *p = 0.0193

### Influence of HIV-positive and HIV-negative plasma exosomes on the phenotypic profile of monocyte subsets

**Fig. 3:**
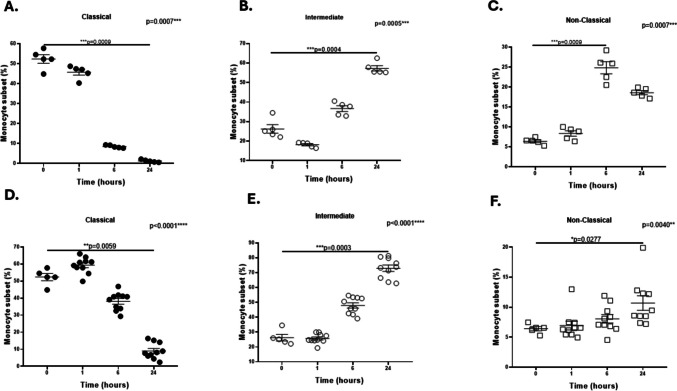
Effects of HIV-negative and HIV-positive plasma exosomes on the percentages of CD14/CD16 monocyte subsets. PBMCs from healthy female donors were incubated with plasma exosomes from healthy female controls (panels **A**, **B**, **C**) or WLWH (panels **D**, **E**, **F**) and the percentages of monocyte subpopulations were quantified via flow cytometry. (A) The percentage of classical subpopulation significantly decreased at 24 h (***p = 0.0009) than in those at 0 h, whereas intermediate and non-classical subpopulations increased significantly at 24 h (***p = 0.0004) or 6 h (***p = 0.0009), respectively, in monocytes exposed to control exosomes (**B**, **C**). The percentage of classical subpopulation significantly decreased (**p = 0.0059), whereas intermediate and non-classical monocytes significantly increased at 24 h (***p = 0.0003 and *p = 0.0277, respectively) in monocytes exposed to HIV-positive plasma exosomes (**D**, **E**, **F**). Data were expressed as mean ± SEM. Each point on the graph represents the average of 3 assays. *p < 0.05, **p < 0.01, ***p < 0.001, ****p < 0.0001; MFI = Median Fluorescence Intensity. The *p*-value illustrated in the right corner of each panel represents the ANOVA test value for the comparison of the four groups

PBMCs from healthy female donors without HIV were incubated with plasma exosomes from WLWH or controls to determine their effects on the percentages of monocyte subpopulations. The percentage of the classical subpopulation significantly decreased at 24 h (Fig. 3A), whereas intermediate and nonclassical subpopulations significantly increased at 24 and 6 h, respectively, in monocytes exposed to HIV-negative exosomes (Fig. 3B and C). However, a significant decrease was observed in the percentage of the classical subpopulation (Fig. 3D), whereas a significant increase was observed in the intermediate and nonclassical subpopulations, of monocytes incubated with HIV-positive plasma exosomes (Fig. 3E and F).

We also compared the percentages of monocyte subpopulations exposed to HIV-negative exosomes with those exposed to HIV-positive exosomes. The percentage of classical monocytes was significantly greater when the monocytes were exposed to HIV-positive exosomes than when they were incubated with HIV-negative exosomes at 1 h (*p* = 0.0007), 6 h (*p* = 0.0007), or 24 h (*p* = 0.0007) (Table 3). Similarly, the percentage of intermediate monocytes was significantly greater at 1 h (*p* = 0.007), 6 h (*p* = 0.003), and 24 h (*p* = 0.0007), whereas the percentage of nonclassical monocytes was significantly lower at 6 h (*p* = 0.0007) and 24 h (*p* = 0.0083) (Table 3).

**Table 3:** Percentages of monocyte subpopulation exposed to HIV-positive and HIV-negative exosomes at different times

Monocytes Subpopulations	Time	Subpopulation % exposed to HIV- Exosomes **(% ± SEM)**	Subpopulation % exposed to HIV + Exosomes **(% ± SEM)**	*P*-Value
Classical	1 h 6 h 24 h	45.74 ± 1.471 8.484 ± 0.3216 1.218 ± 0.2937	59.28 ± 1.478 38.09 ± 1.666 8.982 ± 1.488	*p = 0.0007 *p = 0.0007 *p = 0.0007
Intermediate	1 h 6 h 24 h	18.16 ± 0.572 36.66 ± 1.482 57.30 ± 1.382	25.71 ± 1.017 47.91 ± 1.777 72.91 ± 2.164	*p = 0.0007 *p = 0.003 *p = 0.0007
Non-Classical	1 h 6 h 24 h	8.364 ± 0.6802 24.84 ± 1.517 18.58 ± 0.5190	6.934 ± 0.722 8.076 ± 0.702 10.69 ± 1.213	p = 0.0942 *p = 0.0007 *p = 0.0083

### Differences in the uptake of plasma exosomes from the NC or SNI groups of WLWH according to monocyte subsets

**Fig. 4:**
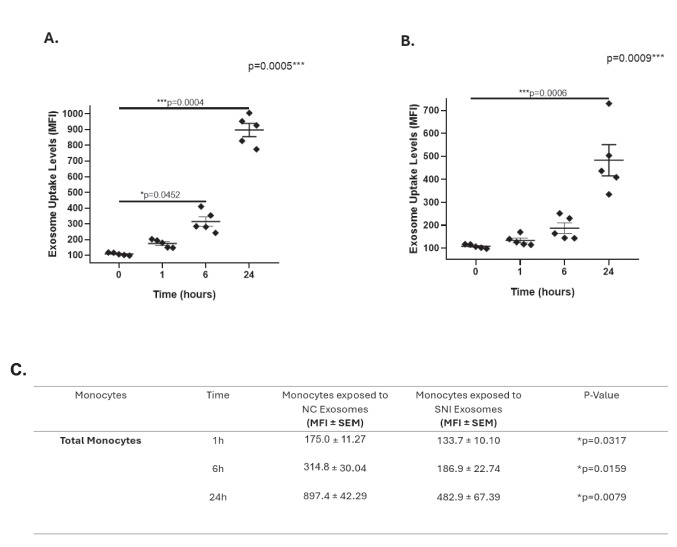
Exosome uptake in total monocytes exposed to NC or SNI exosomes. PBMCs from healthy female donors were incubated with exosomes from WLWH stratified according to NC or SNI and the exosome uptake by total monocytes were monitored via flow cytometry. **A** The exosome uptake significantly increased in monocytes exposed to NC exosomes at 6 h (*p = 0.0452) and 24 h (***p = 0.0004) than in those at 0 h. **B** The exosome uptake significantly increased (***p = 0.0006) in monocytes exposed to SNI exosomes only at 24 h. **C** Table that exosome uptake levels were significantly greater in monocytes exposed to NC exosomes than in those exposed to SNI exosomes at 1 h (*p* = 0.0317), 6 h (*p* = 0.0159), and 24 h (*p* = 0.0079). Data were expressed as mean ± SEM. Each point on the graph represents the average of 3 assays. *p < 0.05, **p < 0.01, **p < 0.001; MFI = Median Fluorescence Intensity. The *p*-value illustrated in the right corner of each panel represents the ANOVA test value for the comparison of the four groups

PBMCs from healthy female donors without HIV were exposed to plasma exosomes from WLWH stratified according to NC or SNI. Our data revealed that exosome uptake levels significantly increased in monocytes exposed to NC exosomes at 6 and 24 h (Fig. 4A) or SNI exosomes only at 24 h (Fig. 4B). Exosome uptake levels were significantly greater in monocytes exposed to NC exosomes than in those exposed to SNI exosomes at 1 h (*p* = 0.0317), 6 h (*p* = 0.0159), and 24 h (*p* = 0.0079) (Fig. 4C).

**Fig. 5:**
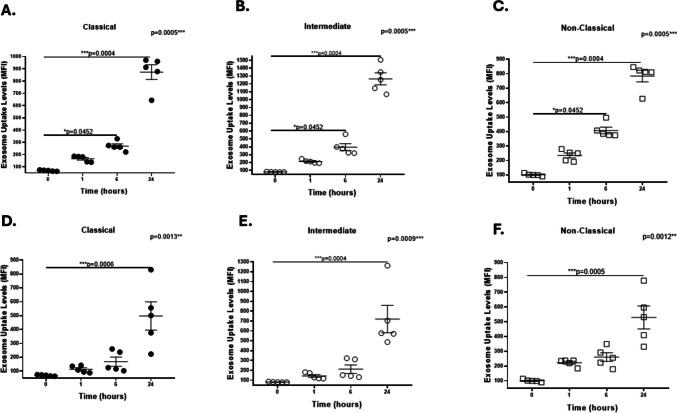
Exosome uptake in CD14/CD16 monocyte subsets exposed to NC or SNI exosomes. PBMCs from healthy female donors were incubated with exosomes from WLWH according to NC (panels **A**, **B**, **C**) or SNI (panels **D**, **E**, **F**) and the exosome uptake in monocyte subpopulations were monitored via flow cytometry. The exosome uptake levels significantly increased in classical (panel **A**), intermediate (panel **B**), and non-classical (panel **C**) exposed to NC exosomes at 6 h (*p = 0.0452, *p = 0.0452, and *p = 0.0452, respectively) or 24 h (***p = 0.0004, ***p = 0.0004, and ***p = 0.0004, respectively). In addition, the exosome uptake levels significantly increased in classical (panel D), intermediate (panel **E**), and non-classical (panel **F**) monocytes exposed to SNI exosomes at 24 h (***p = 0.0006, ***p = 0.0004, and ***p = 0.0005, respectively). Data were expressed as mean ± SEM. Each point on the graph represents the average of 3 assays. *p < 0.05, **p < 0.01, **p < 0.001; MFI = Median Fluorescence Intensity. The *p*-value illustrated in the right corner of each panel represents the ANOVA test value for the comparison of the four groups

Conversely, the exosome uptake levels significantly increased in classical, intermediate, and nonclassical monocytes at 6 and 24 h after exposure to NC exosomes (Fig. 5A, B, C), whereas classical, intermediate, and nonclassical subpopulations exhibited significantly increased exosome uptake levels in monocytes exposed to SNI exosomes at 24 h (Fig. 5D, E, F).

We also compared the exosome uptake levels of monocyte subpopulations exposed to NC exosomes with those of monocyte subpopulations exposed to SNI exosomes at 1, 6, or 24 h. Significantly greater levels of exosome uptake were observed in classical monocytes exposed to NC exosomes than in those incubated with SNI exosomes at 1 h (*p* = 0.0317), 6 h (*p* = 0.0159), and 24 h (*p* = 0.0079). Significantly greater levels of exosome uptake were also observed in the intermediate subpopulation when exposed to NC exosomes than in those incubated with SNI exosomes at 1 h (*p* = 0.0079) and 6 h (*p* = 0.0159) and in nonclassical monocytes at 6 h (*p* = 0.0079) and 24 h (*p* = 0.0159) (Table 4).

**Table 4:** Exosome uptake levels in monocyte subpopulations exposed to NC or SNI exosomes at different times

Monocytes Subpopulations	Time	NC Exosome Uptake **(MFI ± SEM)**	SNI Exosome Uptake **(MFI ± SEM)**	*P*-Value
Classical	1 h 6 h 24 h	166.0 ± 10.86 269.4 ± 17.38 872.4 ± 59.75	113.9 ± 10.84 168.2 ± 33.17 497.4 ± 101.2	*p = 0.0317 *p = 0.0317 *p = 0.0159
Intermediate	1 h 6 h 24 h	213.6 ± 9.86 396.4 ± 43.56 1265 ± 77.01	143.6 ± 13.82 212.2 ± 43.15 720.2 ± 139.8	*p = 0.0079 *p = 0.0159 *p* = 0.0556
Non-Classical	1 h 6 h 24 h	234.6 ± 16.81 407.4 ± 22.91 783.6 ± 39.66	222.2 ± 10.12 259.8 ± 29.06 529.0 ± 77.49	*p* = 0.3810 *p = 0.0079 *p = 0.0159

Additional statistical analyses revealed that nonclassical monocytes exhibited greater exosome uptake than did classical monocytes in the NC (1 h: *p* = 0.0089; 6 h: *p* = 0.0216) and SNI (1 h: *p* = 0.0055) groups, whereas intermediate monocytes exhibited greater NC exosome uptake than did nonclassical monocytes at 24 h (*p* = 0.0034), with no significant differences observed at 6 h or 24 h in the SNI group.

### Influence of the uptake of plasma exosomes from NC or SNI WLWH on the phenotypic profile of monocyte subsets

**Fig. 6:**
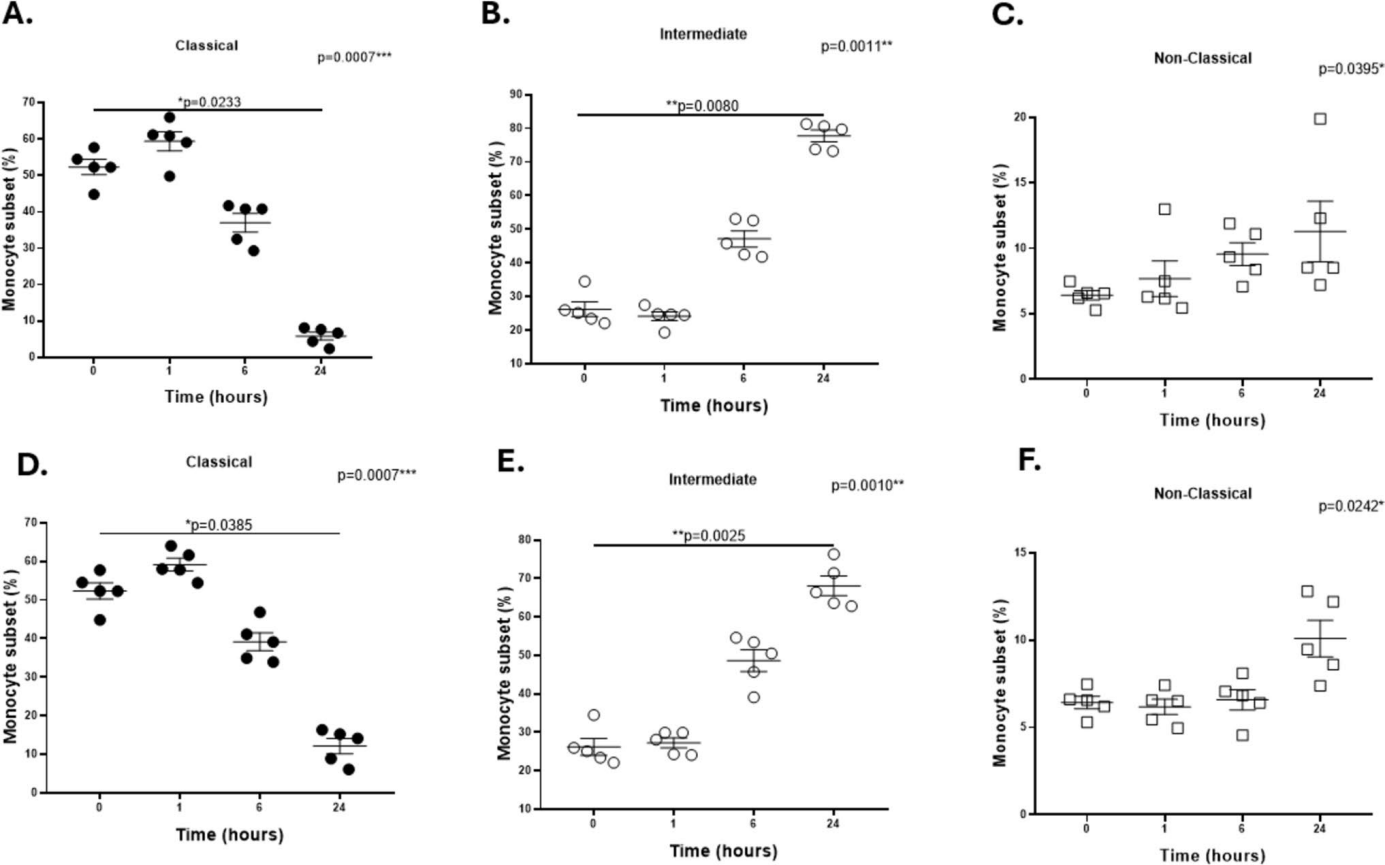
Effects of NC or SNI exosomes on the percentages of CD14/CD16 monocyte subsets. PBMCs from healthy female donors were incubated with exosomes from WLWH stratified according to NC (panels **A**, **B C**) or SNI (panels **D**, **E**, **F**), and the percentage of monocyte subpopulations was monitored via flow cytometry. The percentage of the classical subpopulation in monocytes exposed to NC (panel **A**) and SNI (panel **D**) exosomes significantly decreased at 24 h (*p = 0.0233 and *p = 0.0385, respectively), whereas the intermediate subpopulation exposed to NC (panel **B**) and SNI (panel **E**) exosomes significantly increased (*p = 0.0080 and *p = 0.0025, respectively) at the same time point than in those at 0 h. No differences were observed in the percentage of non-classical subpopulation from monocytes exposed to NC (panel **C**) or SNI (panel **F**) exosomes at 1 h, 6 h, or 24 h. Data were expressed as mean ± SEM. Each point on the graph represents the average of 3 assays. *p < 0.05, **p < 0.01, **p < 0.001; MFI = Median Fluorescence Intensity. The *p*-value illustrated in the right corner of each panel represents the ANOVA test value for the comparison of the four groups

PBMCs from healthy women without HIV were exposed to plasma exosomes from WLWH and stratified according to NC or SNI status to determine their influence on the percentages of monocyte subsets. Compared with that at 0 h, the percentage of the classical subpopulation significantly decreased at 24 h (Fig. 6A, D), whereas the intermediate subpopulation significantly increased at 24 h, when monocytes were incubated with NC or SNI exosomes (Fig. 6B, E). No significant changes were observed in the percentage of the nonclassical subpopulation when monocytes were exposed to NC or SNI exosomes (Fig. 6C, F).

Our results also revealed no significant differences in the percentage of classical monocytes between the NC and SNI groups at each time point (Fig. 6A vs. D). When we compared intermediate monocytes exposed to NC or SNI exosomes (Fig. 6B vs. E), the percentage was significantly greater only at 24 h (*p* = 0.0317). In nonclassical monocytes, the percentage was significantly greater at 6 h (*p* = 0.0159) in cells exposed to NC exosomes than in those exposed to SNI; however, no changes were observed 1 h and 24 h (Fig. 6C vs. F).

## Discussion

Different monocyte subpopulations are associated with HIV neuropathogenesis. Numerous studies suggest that an inflammatory monocyte subpopulation (CD14 + CD16 +) is persistent in the blood circulation of ART-treated PLWH and is associated with HAND by transmigrating across the BBB into the CNS (Buckner et al. 2011; Campbell et al. 2014; Fischer-Smith 2001; Ndhlovu et al. 2015; Valcour et al. 2011; Veenstra et al. 2017, 2019; Williams et al. 2014; Williams et al. 2012, 2013; Wong et al. 2019). Although many studies have reported an expansion of circulating intermediate and nonclassical monocytes in PLWH despite ART, further data are needed to understand the etiology of the CD14 + CD16 + subpopulation that is associated with HAND. Specifically, the factors that are responsible for the variations in the levels of CD14/CD16 monocyte subpopulations in ART-treated WLWH are not clearly understood.

Exosomes released from cells can exert regulatory influences on other cells, such as the modulation of host immune responses and microbial pathogenesis (Bellingham et al. 2012). Their secretion from HIV-infected cells may involve different cargo molecules and responses than those of uninfected cells. Previously, we reported in our HLLC cohort that HIV infection significantly increased the number of plasma exosomes containing reactive oxygen species, HIV-1 Tat, and soluble insulin receptor (Cantres-Rosario et al. 2022). However, their possible role in influencing the phenotypic changes of HIV-uninfected monocyte subpopulations has never been explored. In this study, we investigated whether plasma exosomes from WLWH stratified according to HAND can be internalized by monocytes from healthy female donors and induce changes in their CD14/CD16 phenotypic profiles (including classical, nonclassical, and intermediate subsets).

Our initial results revealed that exosomes from the plasma of WLWH patients or healthy controls were internalized by monocytes from healthy female donors in a time-dependent manner. Differences were observed in the monocytic uptake of exosomes from WLWH patients compared with that from healthy controls. Although monocytes exposed to both groups of exosomes exhibited significantly increased uptake at 24 h of incubation, the exosomes from WLWH increased monocytic uptake at an early time point of 6 h. When we evaluated exosome uptake in all three monocyte subpopulations, we observed an increase in all of the subsets in a time-dependent manner, with the increase being greater at 24 h. However, when we compared monocytes exposed to HIV-negative exosomes with those incubated with HIV-positive exosomes, the classical and intermediate monocyte subsets exhibited increased intracellular exosome levels (MFIs) when exposed to exosomes collected from WLWH (Fig. 2).

These findings suggest the presence of different modified cargos between HIV-positive and HIV-negative exosomes or the presence of different membrane receptors in monocyte subsets that can influence internalization. For example, studies have revealed that exosomes derived from HIV-infected cells rapidly enter recipient cells through the epidermal growth factor receptor (Chen et al. 2018). These receptors are present in peripheral blood monocytes and monocyte-derived macrophages, thus making them susceptible to exosome internalization (Chan et al. 2009; Lamb et al. 2004). Further studies are needed to characterize and identify the differences in monocyte receptors that are responsible for the entrance of HIV-positive exosomes compared with those of HIV-negative controls.

Our findings also support the unique individual functions of classical, intermediate, and nonclassical monocyte subpopulations when exposed to exosomes from WLWH stratified into NC and SNI groups. Our data revealed differences in exosome uptake among all of the monocyte subsets exposed to both NC and SNI exosomes (Figs. 4 and 5). This finding is interesting due to the fact that variations in the functions and levels of monocyte subsets have been identified in PLWH and HAND patients. Veenhuis et al. reported that higher proportions of intermediate monocytes and lower proportions of classical monocytes are associated with poorer cognitive function in U.S. cohorts of virologically suppressed WLWH (Veenhuis et al. 2021). Other studies suggest that the intermediate monocyte subpopulation is more permissive to HIV infection because of relatively higher expression levels of the HIV coreceptor CCR5, and these cells preferentially transmigrate across the BBB via higher surface cell expression of CCR2. Moreover, the cells can transport HIV into the CNS and release viral proteins, cytokines, and chemokines that trigger neuronal damage (Buckner et al. 2011; Rao et al. 2014; Veenhuis et al. 2021; Williams et al. 2012).

We also observed a decrease in the percentage of classical monocytes from 1 to 24 h after exposure to HIV-positive exosomes, whereas the percentage of the intermediate subpopulation significantly increased during the same time period (Fig. 6). This shift in phenotype to a more intermediate state as the number of classical monocytes decreases may suggest a role in the cell differentiation induced by HIV-positive exosomes. Further studies are warranted to elucidate the role of plasma HIV-positive exosomes in the increased proportions of classical and intermediate monocytes and decreased proportions of nonclassical monocytes.

Interestingly, higher percentages of all of the monocyte subpopulations were observed in cells exposed to NC exosomes at 1 h, 6 h and 24 h than in those exposed to SNI exosomes (Table 4). This finding is very important and requires more attention, due to the fact that it may provide insights into an early transitional stage of monocytic subpopulations associated with HAND and possible novel biomarkers in PLWH. These novel results may also help to identify novel targets and mechanisms at early time points in the progression of this condition to prevent or delay the progression to HAND.

Our study has certain limitations, such as the lack of information related to the date when the WLWH who donated exosomes became infected, the small sample size, and the availability of exosomes that solely originated from NC or SNI groups. Additionally, the lack of inclusion of plasma exosomes from men living with HIV (MLWH) represents a weakness of the study, due to the fact that our HLLC is solely comprised of WLWH. However, the results obtained from this study may provide information for future research investigating gender differences between men and women. HAND is a spectrum of neurocognitive dysfunction that also affects PLWH with asymptomatic neurocognitive impairment (ANI). This group is equally important because it is identified as being a transition point between unimpaired and impaired cognition. However, our data support the importance of evaluating the role of plasma exosomes from WLWH stratified according to HAND in the etiology of monocyte subsets and the mechanisms associated with the cognitive impairment that is observed in these patients.

## Data Availability

No datasets were generated or analysed during the current study.
